# Sensitivity to Differences in the Motor Origin of Drawings: From Human to Robot

**DOI:** 10.1371/journal.pone.0102318

**Published:** 2014-07-11

**Authors:** Helena De Preester, Manos Tsakiris

**Affiliations:** 1 School of Arts, University College Ghent, & Department of Philosophy and Moral Science, Ghent University, Ghent, Belgium; 2 Department of Psychology, Royal Holloway, University of London, London, United Kingdom; University of Vermont, United States of America

## Abstract

This study explores the idea that an observer is sensitive to differences in the static traces of drawings that are due to differences in motor origin. In particular, our aim was to test if an observer is able to discriminate between drawings made by a robot and by a human in the case where the drawings contain salient kinematic cues for discrimination and in the case where the drawings only contain more subtle kinematic cues. We hypothesized that participants would be able to correctly attribute the drawing to a human or a robot origin when salient kinematic cues are present. In addition, our study shows that observers are also able to detect the producer behind the drawings in the absence of these salient kinematic cues. The design was such that in the absence of salient kinematic cues, the drawings are visually very similar, i.e. only differing in subtle kinematic differences. Observers thus had to rely on these subtle kinematic differences in the line trajectories between drawings. However, not only motor origin (human versus robot) but also motor style (natural versus mechanic) plays a role in attributing a drawing to the correct producer, because participants scored less high when the human hand draws in a relatively mechanical way. Overall, this study suggests that observers are sensitive to subtle kinematic differences between visually similar marks in drawings that have a different motor origin. We offer some possible interpretations inspired by the idea of “motor resonance”.

## Introduction

In recent years, research on the visual perception both of the performing artist's gestures and movements (in particular in dance and theatre performances [1], [2]) and of static traces of gestures and movement (in particular in drawings and paintings) has grown rapidly. Perception of the artist's movements and gestures, directly or via the traces left behind by them, is an essential part of much of our aesthetic experience and appreciation, because they indicate the way a work of art is or has been created or performed. Generally, our appreciation of works of art would be partly anchored in the very creation or performance of them, irrespective of the particular artistic discipline or medium. “How a musical passage is played, how a monologue is delivered, how a piece of fruit, a tree, or a person is delineated and shaded on canvas, how a dance ensemble spreads apart and gathers together – all such artistic realities depend on the living movement dynamics of the artists creating or performing the work – the composers, painters, choreographers, musicians, playwrights, actors, dancers, sculptors. Those dynamics are naturally embodied in the work itself.” [3] The various forms of art would thus embody the different kinetic dynamics of their creation, and these kinetic dynamics would constitute the basis of the aesthetic appreciation we have of works of art. Works of art, be they temporally defined as in music or spatially defined as in drawing, would depend on the living movement dynamics of the artists creating or performing the work. These dynamics are clearly embodied in the work itself in the case of, e.g., dance, but would also be embodied in a drawing or a painting. It is this embodiment of the dynamics of the creation that would give a work of art a certain qualitative character.

Our focus is not on this latter claim, but on the idea implied in it that an observer is sensitive to the dynamics of the static traces of drawings or paintings that embody the creation process. In line with the embodied cognition approach [4], [5], and since the creative process is characterized by particular kinetic or motor dynamics, this would imply that we have to investigate the role of the motor body not only in art practice, but also in the perception of works of art.

Thinking of the hand of an artist in drawing, one can ask how the beholder copes with the static traces of gestures of the draftsman. In his analysis of drawing, art historian David Rosand takes into consideration the time it takes to draw a line and the time it takes to “read” or respond to that line [6]. Elaborating on the idea that we rehearse the artist's gestures internally and follow their rhythms through space and thus through time, Rosand offers an intriguing analysis of how beholders respond to the act of drawing in the static traces of the drawing. Lines in drawings are essentially traces of the movement of the hand, the arm or the full body. Since a drawn line would recall the process of its becoming through the act of drawing, it invokes a range of kinesthetic experiences and makes us participants in the act of drawing. “As the direct record of motions of the body, a drawing inevitably takes us back to the drawing hand, to the body of the draughtsman, in a kinaesthetic circuit.” [7] (p. xii) According to Rosand, when the beholder retraces the activity of drawing in her or his imagination, she can participate in the experience of drawing and appreciate the work.

However, results from experimental research in this relatively new field of interest have remained rather scarce. Recently, Freedberg and Gallese [8] claimed that the mirror neuron system could explain a sense of inward imitation of the observed actions of others in pictures and sculptures that represent or depict movement. The idea that neural and cognitive systems contributing to action production are also active during the observation of others' actions [9]–[15] has led to many proposals concerning the functional role of so-called ‘mirror systems’ in action perception and action understanding [16]–[19]. Freedberg and Gallese [8] apply this idea of “motor resonance” to works of art that represent movement. Battaglia and colleagues [20] show clear motor correlates of the relationship between the aesthetic quality of a work and the perception of implied movement within it, and it is likely that these responses are not restricted to strictly representational art, i.e. to realistic depictions of movement [21]–[22].

Moreover, the idea of “motor resonance” may not only be applicable to the representation of movement in works of art, but also to the traces of the creation process of the work. Freedberg and Gallese claim that observers often “feel a form of somatic response to vigorous handling of the artistic medium and to visual evidence of the movement of the hand more generally” [8] (p. 202) and Gallese [23] conjectures that in observing the graphic traces of an artist's gesture, the same motor centers required for producing the traces are active in the observer. “Our proposal posits that even the artist's gestures producing the art work can induce an empathic engagement of the observer, by activating the simulation of the corresponding motor programme. The marks on the painting/sculpture are the visible traces of goal-directed movements, hence in principle capable of activating the somatotopically relevant motor areas in the observer's brain, as suggested by the mirror neuron research.” [23] (p. 460) Studies indirectly suggesting that this is the case, show that motor simulation can be induced in the observer's brain also when what is directly observed are the static graphic traces produced by the action, such as a letter or a graphic stroke, and not the action itself [24]–[26]. A static form would activate the relevant motor codes for producing the form, and these motor codes would lead to a prediction of the resulting form. As such, handwritten letters are static stimuli in which movement is ascribed long after the action has happened. The brain thus makes a reconstruction of the action on the basis of static information [25]. Therefore, it is suggested that in the perception of a static form which is the trace of human movement, a simulation takes place of the dynamical processes that gave rise to it.

Freedberg & Gallese [8] stress the goal-orientated aspect of our embodied response to the static traces of an artist's gesture. In general, what seems to be crucial in action observation is that actions are goal oriented, rather than that they are performed by a human (or biological) actor. An fMRI-paper by Gazzola and colleagues specifically addressed this issue [27]. The results showed that the mirror system was activated strongly by the sight of both human and robotic hand actions, with no significant differences between these two agents (but see [28]). Indeed, robotic hand actions can be functionally and qua overall motor embodiment similar to human hand actions. Thus, what seems to matter, is not the nature of the agent as such (biological or mechanical) or its visual appearance, but the specifics of its motor embodiment (functionally similar to ours or not). What enables an observer to understand the intended goal of an observed action, would be the shared (by agent and observer) embodiment of the intended goal. In brief, the motor behaviour on the basis of which goals are reached should be sufficiently similar. Therefore, it is supposed that an observer is capable of mirroring observed motor behaviour of an agent that is functionally sufficiently similar. Mirroring would not take place when observing actions executed by agents with which the observer does not share the same motor functionality. Therefore, the movements of many (but not all) mechanical agents would not lead to motor understanding in a human observer.

A study by Umiltà [29] and by Umiltà and colleagues [30] explored if the observation of the visible consequences of an artistic gesture evokes a cortical representation of the motor act that has generated it. In the first condition, subjects observed a photograph of a Lucio Fontana painting with one, two or three vertical cuts made in a white canvas. A control group observed similar images (same shape, colour, position and direction of the cut) but artificially produced, i.e. digitally done with a computer (not to be confused with images e.g. made by a computer controlled robot). They found that stronger mu rhythm suppression was evoked by the observation of an original work of art that consists of traces of the artist's gestures (cuts), and less suppression by its artificial reproduction (lines) that is not the result of any real-world movement. Thus, in spite of a certain similarity between the images, an image of cuts evokes more cortical motor activation in the brain of the beholder (as exemplified by stronger reduced mu rhythm suppression) than an image of lines reproducing the cuts.

As is clearly visible on the first figure provided in the study [30] an important dissimilarity between the two conditions was that the lines of the artificial reproduction of the cuts did not reproduce the changing width of the cut in the canvas. As implied by the study, this is an important visual cue for perceiving how the cuts have been produced. In the absence of changing width, a line does not look as the result of a movement. The artificial character of the artificially reproduced cuts, and consequently that they were not produced in a motor way, was thus clear. This suggests that the observer is sensitive to whether a form is produced on the basis of (human) movement or digitally produced, and thus not having a motor origin.

In our study, we further explored the sensitivity of an observer to the motor origin of static traces, i.e. we concentrated on the question whether an observer is also sensitive to differences within the motor origin condition. In extending the studies by Umiltà and colleagues, we aimed to test if an observer is sensitive to the differences, not between motor and non-motor origin, but between different motor origins: human and robot. We thus explored what happened, first, when stimuli were produced by different draftsmen (human and robot) that physically produce lines on the basis of different motor repertoires. Second, and since the images used in the studies by Umiltà and colleagues exhibited salient cues as to their motor origin, we also explored the situation in which such salient cues for discrimination between different motor origins are absent and only more subtle kinematic cues are present. In summary, this study explores how sensitive we are to differences in traces resulting from differences in (bio)mechanics and kinematics: are we only sensitive to obvious traces resulting from differences in kinematics or are we also sensitive to more subtle traces resulting from differences in kinematics?

## Design and Materials

In order to answer the question if and how sensitive an observer is to differences between different motor origins, the stimuli used in the experiment are drawings produced by different draftsmen (human and robot). More in particular, three different agents produced a similar series of drawings. Two of the three agents were humans, the third agent was a robot. Two of the three agents had a more mechanical way of drawing (the robot and a computer artist) whereas the third agent (a sculptor) drew, not in a mechanical, but in a natural way. “Way of drawing”, or “style” (in particular artistic style) is a difficult notion to define since it is dependent on a wide variety of parameters. In this experiment, however, the drawings mainly differ with regard to the kinematic parameters of the drawn lines, reflecting the idea that drawings are characterized by the kinetic dynamics that have produced them. It is well known that biological and non-biological motion have very different acceleration and velocity profiles [32]: the most important kinematic parameters resulting into different line dynamics (e.g. regularity of the lines) are velocity and acceleration. Overall, the robot was drawing much faster than the humans, and mostly (except for one drawing) operated at constant velocity. When changing direction of the line, the robot halted to turn and once turned, continued its way again at constant velocity. Humans, in contrast, constantly accelerate and decelerate when drawing. Moreover, they do not necessarily halt but rather decelerate when changing direction of the line. This results in lines with a more fluid and natural, but also more sloppy or less regular look or style, whereas robot lines manifest a more regular, but also more rigid, neat and mechanical style. The differences, however, are subtle, because the algorithms that controlled the robot were written especially in order to draw in a less machine-like way [33]. Nonetheless, this did not prevent that repeated elements within one robot drawing are identical in overall size and form.

Another important feature of the style or way of drawing is the pressure with which lines are drawn. Together with the kinematic parameter of velocity, fewer pressure by the robot resulted in less dense lines for the robot drawings. The human hand exercised more pressure on the pen and was drawing slower than the robot, such that the ink of the human drawings was denser, which resulted in darker drawings. Because the density of the line is a style feature that not only depends on the kinematics of the movement, we controlled for this in the design. To ensure that participants would not use systematic differences in overall darkness of the drawings as a cue for discriminating human and robot drawings, midtones of the robot drawings were adjusted such that they matched the human drawings qua darkness. In sum, it was mainly differences in velocity and acceleration (or lack thereof) that resulted in style differences between human and robot drawings.

Drawings ranged from simple, single lines to more complex drawings. The non-figurative, abstract drawings were not representing anything. A series of 20 drawings was produced by a robot (programmed by a new media artist). Two different artists then copied this same series by hand and each robot drawing was thus copied twice (by two different hands), resulting into three similar series of 20 drawings each. The sculptor was asked to copy the drawings because of his trained hand and spatial insight, and since, more generally, sculptors often are accomplished draftsmen exhibiting fluid, natural drawing styles. The computer artist was asked to copy the drawings because his many sketchbooks from over the years exhibit a style of drawing that struck as pen plotter-like, in line with his long-standing practice as a computer artist. A pen plotter is a computer printer that draws lines with one or more automated pens attached to arms that move mechanically over the paper, and the resulting drawings openly look mechanical. We asked the computer artist to copy the robot drawings because we wanted to include an intermediate case between the robot and the human drawings, or rather, between the drawings with a mechanical style and with a natural style. We included his drawings in the experiment in order to test if participants would hesitate about the motor origin of the drawing, i.e. hesitating between a human and a robot origin. A third artist, a new media artist, was asked to program his drawing robot with self-written algorithms, which were developed during an artistic research process and intended to make robot drawings look less mechanical and more humanlike. In that way, we wanted to prevent that, overall, the robot images would show too patently their mechanical-digital origin.

**Figure 1:**
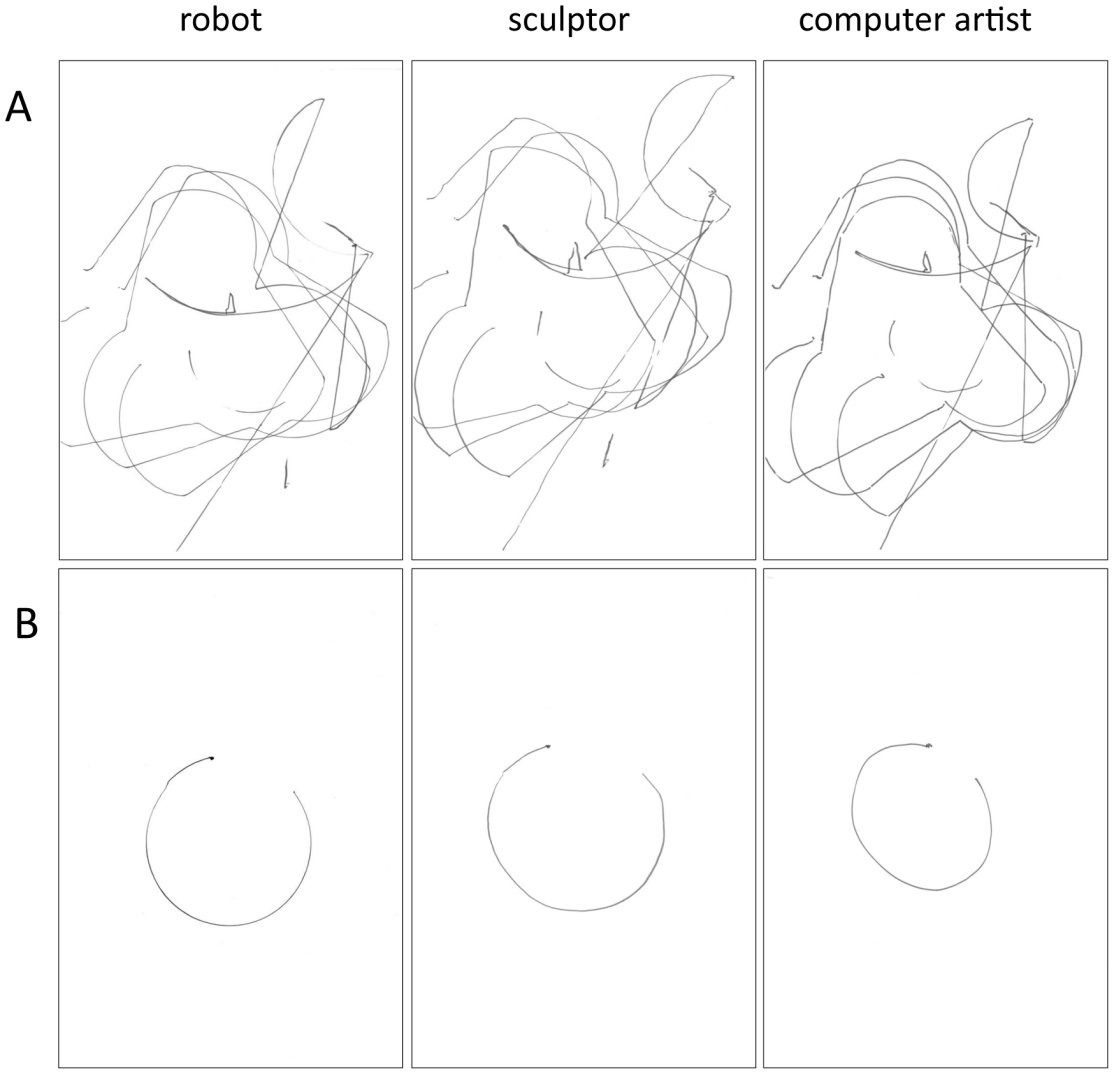
Sample of drawings with salient cues. Both panel A and B show “salient kinematic cue” drawings. From left to right: robot drawing, sculptor drawing, computer artist drawing.

**Figure 2:**
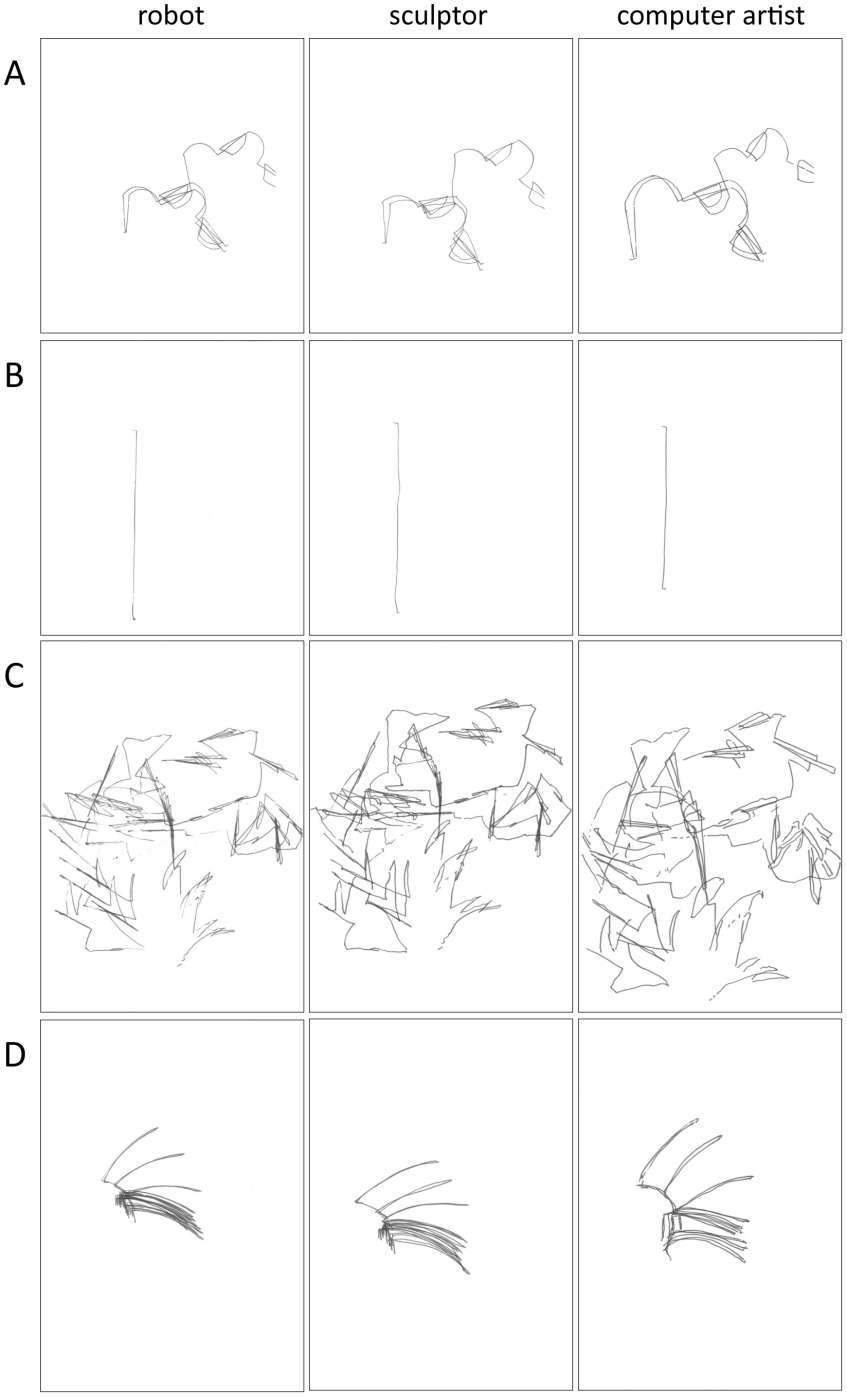
Sample of drawings without salient cues. All panels show “subtle kinematic cue” drawings. From left to right: robot drawing, sculptor drawing, computer artist drawing.

At the same time, and in order to explore the role of salient versus subtle kinematic cues for discrimination between different motor origins, the drawings were also categorized into drawings containing salient kinematic cues and drawings only containing subtle kinematic cues. Thus, a first category of drawings contained salient kinematic cues on the basis of which observers could easily visually judge whether the drawing was made by a human or a robot. These salient kinematic kinematic cues consisted of well-formed circles or fragments of circular forms of 180° or more. Since in drawing position, the human wrist cannot rotate around its axis (i.e. maintaining a constant radius) for approximately more than 180°, the presence of circles and fragments of circles of 180° or more served as salient kinematic kinematic cue for discriminating human versus robot drawings. We relied on the well known fact that even for the most talented of artists, one of the most difficult things to draw is a circle. For example, Giorgio Vasari [31], in the sixteenth century, relates that when the Pope sent a messenger to Giotto, asking him to send a drawing to demonstrate his skill, Giotto drew a circle (in red paint), and this circle was so perfect that it seemed as though it was drawn using a compass. Making use of the circle for discriminating between artist and machine or mechanical device is thus not new, and seems a robust method. This category of drawings containing salient kinematic cues for discriminating between human and robot origin is thus defined on the basis of movements that are anatomically/kinematically impossible for the a human hand, but possible for the robot, underscoring the emphasis on the involvement of the motor dimension. Since in drawing position, the human wrist cannot rotate more than 180° around its own axis, whereas the robot could rotate a 360° around its own axis, this resulted in well-formed circles or fragments of circles of 180° or more in the case of the robot, and more sloppy circles or fragments of circles of 180° or more in the case of the human hand. Curvilinear fragments or circular fragments smaller than 180° were copied much more adequately by the human hand and could not serve as salient kinematic cues, whereas straight lines were in both the human and the robot drawings not straight as if drawn along a ruler. As such, they could not serve as salient kinematic cues for discriminating human from robot drawings. The computer artist and the sculptor were asked to copy the drawings as accurately as possible, implying that the salient kinematic cue of circular forms or fragments thereof would be respected and thus included. The problem of drawing circles or large fragments of circles (i.e. requiring a shift of arm position) is a notorious one in drawing practice. Both artists (and more generally anyone acquainted with drawing as a practitioner or as a beholder) were well aware that regular circular forms that exceed the rotation possibilities of the wrist are a challenge when drawing, and also perceptually conspicuous for the onlooker, because of the salient kinematic differences between free hand drawing and mechanical drawing (e.g. using a compass, or executed by another mechanical device such as a robot that is able to rotate around its axis). In what follows, we therefore label the robot and the human drawings containing circular forms or large fragments thereof as containing “salient kinematic cues” (see Fig. 1) and drawings without these elements as containing “subtle kinematic cues” (see Fig. 2). Thus, by making a category of drawings containing a notoriously difficult element for humans to draw (resulting in visually conspicuous differences between robot and human producers), we wanted to make an improvement compared to the study by Umiltà and colleagues, since now two categories of drawings are presented, one with salient kinematic cues for discriminating between motor origins of the drawings, and one only containing subtle kinematic cues.

The robot (developed and programmed by the new media artist) produced a series of 20 drawings, and this series was then copied by hand by two artists, a sculptor and a computer artist (cf. supra). The material used (Faber-Castell PITT artist pen, soft tip, with cold grey Indian ink, on A3 Steinbach Aurora drawing paper of 200 g) was the same for the three agents. The algorithms for the robot were developed in the course of an artistic PhD-project (Poetic Machine, 2006–2012 [33]). At first sight, and although mechanically produced, the resulting drawings do not openly look like robot drawings. This was intended to be so, in order to approach the look of human drawings as closely as possible and thus to present robot drawings that were visually as similar as possible to human drawings.

The original drawings on A3-format were first digitalized by scanning them in high resolution (7015×9921 pixels). In order to present them in the Presentation 14.9 software, the images were resized to 702×992 pixels. These were displayed on a screen with a resolution of 1680×1050 and 20 inch diagonal (99.06 ppi pixel density), resulting in images of 7.09×10.01 inch or 18.01×25.43 cm. Since the test room does not let in any daylight, brightness was set a 100 cd/m2, matching the visual brightness of the original drawings in similar lighting conditions. As for the contrast, since a higher contrast ratio will show more tonal gradations, we opted for a relatively low contrast ratio, around 500∶1, in view of the fact that we wanted to control for differences in tonal gradation. Ambient light was kept constant through the use of ceiling light fittings (4×50 W). Participants sat comfortably on a chair, and viewing distance was kept constant and comfortable across all participants at 60 cm.

### Participants

12 naïve (i.e. not educated in the domain of fine arts or art history) volunteers participated in this experiment after giving their written informed consent (8 female, mean age: 33.6) with normal or corrected-to-normal vision. The study was approved by the Departmental Ethics Committee, Department of Psychology, Royal Holloway, University of London.

### Procedure

On each trial, participants were presented with one drawing (with salient kinematic cues for discriminating or with subtle kinematic cues only) that was produced by the robot, the computer artist or the sculptor, resulting in a 2×3 within subjects design, and were asked to judge in an unspeeded way if the drawing was made by a human or by a robot. The number of drawings with salient kinematic cues was 6 out of 20 for each producer (robot, computer artist, sculptor) and the number of drawings with subtle kinematic cues only was 14 out of 20 for each producer. In total, each participant was presented with the same series of drawings: 24 drawings with salient kinematic cues and 56 drawings without subtle kinematic cues only. Since we wanted to have an equal number of presentations of stimuli from robots and humans, each robot drawing was presented twice in the series, resulting in 80 drawings. The series of 80 drawings thus contained 20 times 4 very similar-looking drawings, two by a robot (twice the same), two by a human (by two different draftsmen) (see Fig. 1 en 2, robot drawings shown once).

Participants were presented with the 80 drawings, one by one and in random order across participants, presented in Presentation 14.9, on a Samsung SyncMaster 2043 BW screen. Participants were instructed to judge each time if the drawing was made by a human or by a robot, and to make their choice by pressing the corresponding key on the keyboard. The exact wording of the instruction was: “You will see, one by one, a drawing on the screen. The drawing is made by a robot, or by a human. You decide, on the basis of a thought or a feeling or an intuition, if the drawing you see is made by a robot or by a human hand by pressing the corresponding button. There is no need to hurry, you can take all the time you need before deciding.” No more instructions were given and once they had pressed a key, the next image appeared. Participants were not informed about the visual or functional properties of the robot and did not know that two different human hands were involved or that this was a collaboration with artists. They were not informed about who copied whom, or what the proportion of human and robot drawings was, and they were not given any feedback on their performance during the course of the experiment.

The set-up of the study, the nature of the drawings, and the scarcity of information about the agents were such that apart from a number of drawings containing the mentioned salient kinematic cues, there were no other salient cues as to the producer of the drawings.

## Results

We wanted to test if an observer would be able to discriminate between the human and the robot producers (1) when there were salient kinematic cues for discriminating between the drawings and (2) when there were only subtle kinematic cues. We hypothesized that participants would be able to attribute the drawing to the correct motor origin (human or robot) when salient kinematic cues were present, but in the presence of subtle kinematic cues only, that they would become confused about the motor origin of the drawings made by the computer artist (drawings with a human motor origin but a rather mechanical drawing style).

We measured participants' accuracy, expressed as a % correct rate, in correctly recognizing the origin of the drawing (i.e. human or robot). Across all participants, there was an above chance performance, with 70% of the drawings being attributed to the correct producer (human or robot). 10 out of 12 participants performed above chance level (binomial test, H_0_: π = .5, 1-α = .95, with Yates' correction for continuity). The score of the two participants performing at chance level was 58.75% of correct answers (binomial test, H_0_: π = .5, 1-α = .95, with Yates' correction for continuity, p = 0.0735).

Because we were interested in the role of kinematic cues and the role of the producer of the drawing, the mean correct rate per condition was submitted in a 2×3 repeated measures ANOVA with the factors of type of drawing (“salient” versus “subtle”) and producer of the drawing (robot, sculptor, and computer artist). There was a significant main effect of kinematic cue (salient versus subtle) on participants' performance (percentage of correct answers): F(1,11) = 17.498, p = .002. Overall performance was better when judging drawings containing salient kinematic cues (“salient”) (83.3%) than when judging drawings containing only subtle cues (“subtle”) (64.4%). This latter score is nonetheless still above chance level (64.4%, binomial test, H_0_: π = .5, 1-α = .95, with Yates' correction for continuity, p = .0026).

The main effect of producer was not significant (F(2,22) = 2.189, p = .136), but the interaction between category of drawing (“salient kinematic cues” versus “subtle kinematic cues”) and producer was significant (F(2,22) = 4.608; p = .021). To investigate the origin of this interaction, we performed planned comparisons. We also calculated an estimate for proportion of variance explained by the different factors. More in particular, we calculated the values for Cohen's f, based on the measurement for explained variance partial eta squared (*f^2^ = η^2^/ 1−η^2^*). Since *η^2^* is not a standardized measure, we compare Cohen's f-values. The factor category of drawing (i.e. with salient kinematic cues or with subtle kinematic cues only) has f = 1.26 (η^2^: .614), the factor producer (robot, sculptor or computer artist) f = .45 (η^2^: .166), and for the interaction factor (type of drawing × producer) we have f = .65 (η^2^: .295). These are all large effect sizes.

We did not expect any significant difference between producers in the “salient kinematic cues” condition, since participants could easily rely on these salient visual cues across the three producers. Indeed, the percentage of correct answers was high for all three producers: sculptor (M = 80.6, SD = 17.1), computer artist (M = 77.7, SD = 16.3) and robot (M = 87.5, SD = 15.7). Paired-samples t-tests of mean differences showed that for drawings containing salient kinematic cues, there was no significant difference in means of correct answers between the different producers, i.e. for drawings by the sculptor and the computer artist (*t*(11) = .417, p = .685, 2-tailed), for drawings by the robot and the sculptor (*t*(11) = .933, p = .371, 2-tailed), and for drawings by the robot and the computer artist (*t*(11) = 1.790, p = .101, 2-tailed)).

**Figure 3:**
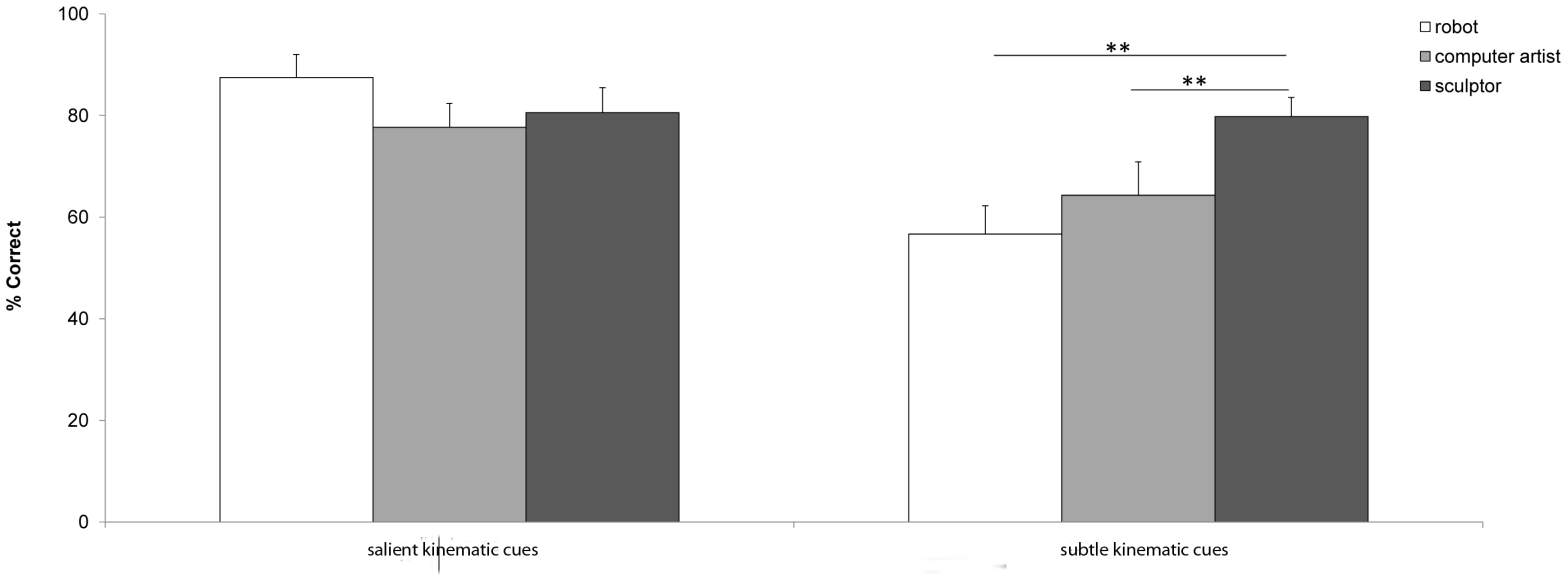
Percentage of correct attributions. Percentage of correct attributions of drawings produced by the robot, the sculptor and the computer artist, for drawings with salient kinematic cues and with subtle kinematic cues. Error bars depict Standard Error of Means.

Therefore, we were further primarily interested in the “subtle kinematic cues” drawings, and further investigated with planned comparisons between the three producers. For the “subtle kinematic cues” drawings, there was a significant difference in means between robot drawings and drawings made by the sculptor (M = 79.8, SD = 13.1) (t(11) = −3.221, p = .008, 2-tailed), and between drawings made by the sculptor and by the computer artist (t(11) = 2.614, p = .024, 2-tailed), whereas there was no significant difference in means of correct answer between robot drawings (M = 56.7, SD = 19.3) and drawings made by the computer artist (M = 64.3, SD = 22.9) (t(11) = −.938, p = .368, 2-tailed) (see Fig. 3). Moreover, all three means are above chance (robot drawings, 56.8%, p = .0069, computer artist drawings, 64.3%, p = .0026, sculptor, 79.8%, p = .0026, all binomial test, H_0_: π = .5, 1-α = .95, with Yates' correction for continuity).

## Discussion

The purpose of the study was to test if an observer is sensitive to differences in static traces of drawings that have different motor origins (a drawing robot, a human with a natural drawing style and a human with a less natural and more mechanical drawing style). We tested this by checking if participants could attribute drawings to the correct producer. We hypothesized that participants would be able to correctly attribute the drawing to a human or a robot origin when salient kinematic cues are present, and when only subtle kinematic cues are present, that the observer would still be able to recognize the hand of the sculptor as human, but would be confused about the drawings made by the computer artist. Overall, this would suggest what the role of salient kinematic cues is versus the role of subtle kinematic details.

As expected, drawings containing salient kinematic cues were more often judged correctly than drawings with only subtle kinematic cues. For “salient kinematic cues” drawings, participants perform equally well for the three producers and obtain high scores for all producers. The salient kinematic cues we used turn out to be very reliable when it comes to distinguishing traces by a human hand from traces by a machine or a robot. A possible explanation for these (equally) high scores for the three producers, could be that observers rely on their knowledge or prototypical view of robots and humans. Well-formed circular shapes are visually conspicuous and in accordance with the prototypical view of robots as agents working neatly, accurately and in a mechanical way. This stands in contrast to the more natural and (geometrically) sloppier way of drawing by a human hand, which is not able to draw without aids well-formed circular shapes or large fragments thereof. Knowledge about the mechanical movement of robots could thus have informed their decisions, which was supported by visual expertise in distinguishing regular circles or large circle fragments from irregular ones.

Since we were interested in cases in which the difference between robot and human drawings is visually harder to detect, i.e. when only subtle kinematic cues are present, and on the basis of the significant interaction between producer and type of drawing, further analysis was focused on the “subtle kinematic cues” drawings. It is primarily with regard to subtle kinematic features, caused by differences in drawing movements, that the “subtle kinematic cues” drawings differ. It is here that the advantage of the collaboration with the new media artist who programmed the robot became apparent: in combination with the contingencies associated with drawing in the real world (irregularities in the paper or the drawing surface, physical properties of ink, pen and paper), the drawings made by the robot exhibited (on purpose of the programmer) hesitations and flaws, and the robot did not produce straight, neat lines that did not vary in thickness or width at all. This means that in the “subtle kinematic cues” drawings, well-formedness of the elements could no longer play a decisive role in discriminating traces by a human hand from robot traces. Differences between the motor origins of the drawn traces could only be detected on the basis of subtle differences in the kinematic parameters. Interestingly, participants scored very well for the sculptor drawings, but significantly less well when judging the drawings made by the computer artist (see Fig. 3). Although participants still perform above chance in correctly attributing the computer artist drawings to a human hand, there is a large drop in percentage of correct attributions, showing that participants have more difficulties in detecting the subtle cues for attributing the drawing to a human hand in the case of the drawings made by the computer artist. In addition, the performance for the drawings made by the sculptor was significantly better compared to the performance for the drawings made by the computer artist and the drawings made by the robot. Presumably, this is because participants were able to use the kinematic parameters of the drawings by the sculptor as reliable indicators for their motor origin. In short, they used fluidity and naturalness of the drawn lines as indications for human movement. Together, these two observations confirmed our idea that there was a significant difference between the computer artist's drawing style (cf. its resemblance to plotted lines) and the drawing style of the sculptor (cf. its fluid and natural way of drawing).

A study by Cross and colleagues [34] shows that the action observation network (AON) is sensitive to a broader range of action features beyond those that are simply familiar. In that study, reactions to observing videos of human/natural versus robot movements (both performed by a human as well as by a virtual Lego robot) were compared. The study showed that the action observation network responded more robustly to robot-like motion (for both human and Lego robot forms). This is consistent with the findings of the present study about the traces of human versus robot movement in the sense that it is not the agent performing or creating that matters most, but the style of movement/drawing, i.e. the kinematic details or the kinetic dynamics of the lines. Cross and colleagues challenge the idea that the action observation network is only responsive to human agents or only to actions that are familiar (cf. the dominant familiarity hypothesis). Instead, there appears to be a dissociation between how well participants think they can perform an action and activation of the action observation network. When participants were asked to rate their ability to reproduce the dance movements, they rated the robotic movements as more difficult, and no main effect of agent (human or Lego robot) was present. This suggests that sharing the same motor repertoire is crucial, but it also suggests that motor resonance is more complex than simply a heightened activation of the action observation network. In order to investigate if the performance by participants relies on the activation of the action observation network or on the activation of the mirror neuron system, or if it relies on the internal rehearsal of the (implied) movement observed, e.g. based on the degree of prediction error and thus mismatch instead of match, further studies are needed. What is important in the study by Cross and colleagues, however, is the uncoupling of agent and movement, something we took into account in our design by including the drawings made by the computer artist. Our study suggests that this uncoupling does not only happen in the case of observing live movements (as in Cross et al.), but also when observing the static traces of movement. Sensitivity for differences in kinetic dynamics would thus transfer from live movement to traces of movement.

The difference in motor dynamics between the three agents is reflected in kinematic details of the lines. Moreover, in the absence of salient kinematic cues, observers can only rely on subtle kinematic details in order to judge the motor origin of the drawings (robot or human). Figure 2 shows that these differences in drawing style are indeed very subtle ones, but nonetheless must be responsible for the significant difference in percentage of correct attributions. Since the kinematic features of the lines are indicators for the movements that have produced the lines, we should look at theories and results that point into the direction of an (implicit) recognition of the movement involved.

Our results extend the main findings of the study by Umiltà and colleagues by showing that observers are not only able to detect traces resulting from movement versus “traces” not resulting from movement, but that they are also sensitive to differences between several motor origins. In addition, we controlled for salient cues and focused on the effect of more subtle kinematic features of the lines.

It has been proposed that our motor system is geared up to execute observed movements, i.e. that observing an action would excite the motor programs used to execute the action oneself [35]. A study by Kilner and colleagues [32] showed that the observation of another human making incongruent arm movements significantly interfered with the execution of arm movements, but not when incongruent robot movements were observed. These results suggest that there is a distinction between observing human and robotic movements in terms of this interference effect. Biological (human) and non-biological (robot) movements would be two types of movements, processed by distinct neural systems. Kilner and colleagues say that many aspects of human movement could have caused interference (in the congruent condition), including the velocity profile of the movement, the bodily posture, or the presence of bodily, head, or facial features of the human. Our study, together with the results from Cross and colleagues [34], suggests that especially the kinematics of movement play a role in the sensitivity for the motor origin of movement. Thus, the two types of movement (biological and robot) are primarily distinguished on the basis of the kinematics of the (traces of) movement. The representation of a human or more generally of the executor of the movement is not necessary in order to be able to discriminate between different motor origins.

Since the mirror system might have evolved in the context of action understanding and empathy [36] it is not unlikely that mirror neurons play a role in the better recognition of the sculptor's traces as traces of human movement, whereas perceptual sensitivity for more mechanical traces is less accurate. Although “motor resonance” as such does not necessarily imply a better recognition of human movement versus robot, Calvo-Merino and colleagues [37] have shown that we do not only understand actions by visual recognition, but also motorically. Mirror circuits have a purely motor response over and above visual representations of action. This would imply for our study that robot drawings are primarily judged on a visual basis, implying less motor response than the drawings that are made by a naturally drawing human hand. The latter would not only be recognized on the basis of a purely visual strategy, but the kinematic details would also lead to a motor understanding of the movement implied. This additional role of motor understanding in the case of human traces could be a factor in explaining the higher scores for the drawings made by the sculptor, and the more hesitant attitude towards drawings by the computer artist (and to a lesser degree also the robot). If we don't appeal to an additional motor understanding, it would be hard to explain the possible basis for the difference in scores between human drawings, robot drawings and the in between drawings.

A final point takes into account the so-called “uncanny valley” phenomenon. Saygin and colleagues [37] also looked at the effects of an android (biological appearance but mechanical movement) on brain activity, in particular in the action perception system. The expectation that an agent that looks human also moves biologically, is violated here, and would be a factor in the explanation of the “uncanny valley” phenomenon. The “uncanny valley” refers to the point where the positive correlation between the human appearance of a robot and the feeling of familiarity of humans toward the robot suddenly breaks down when the robot's appearance becomes very human-like, leading to a feeling of uncanniness [39]. Saygin and colleagues [38] observed similar suppression effects for the human and the robot, and stronger for the android, especially in a key node of the action perception network (anterior intraparietal sulcus). It is very difficult to test if something similar to the uncanny valley phenomenon is also possible in the case of static traces, because the required mismatch between appearance of the agent and movement is not realizable. What is present, however, in our study, is an attempt to “humanize” the robot lines on the basis of algorithms that result in less rigid robot movements, and a reverse attempt to “roboticize” the lines of one human agent by asking someone with a more plotter-like drawing style to produce a series of stimuli (without however instructing the artist to draw like a robot). Therefore, in both cases, an effect of uncanniness might be involved, since the robot drawings are not robot-like at first sight, and the computer artist drawings might not be straightforwardly human at first sight. This may be an alternative way of explaining why observers in the “subtle kinematic cues” condition generally perform well for the unambiguously human drawings by the sculptor but get confused by the robot and computer artist drawings. As both Ernst Jentsch and Sigmund Freud observed, ambiguity and doubt if something is living or dead, or moving because it is alive or mechanically is one of the key features of the uncanny [40]–[41]. As Freud remarks: “Jentsch has taken as a very good instance ‘doubts whether an apparently animate being is really alive; or conversely, whether lifeless objects might not be in fact animate’; and he refers in this connection to the impression made by waxwork figures, ingeniously constructed dolls and automata. To these he adds the uncanny effect of epileptic fits, and of manifestations of insanity, because these excite in the spectator the impression of automatic mechanical processes at work behind the ordinary appearance of mental activity.” [41] (p. 226) It is not sure that the “uncanny valley”-explanation and motor understanding as addition to visual understanding are mutually exclusive in explaining the observed differences in scores, since both focus on movement as an essential element in our recognition of movement (or traces of movement) or lack thereof.

## Conclusions

Starting from the idea of “motor resonance”, i.e. the idea that in the perception of static traces of human movement, a simulation takes place of the dynamic processes that gave rise to it, we focused on the role of the kinetic dynamics or the kinematic features of lines in drawings, and on the observer's sensitivity to differences in motor origin. In line with the finding that it is not the nature of the agent as such or the mere visual appearance of its product, but its motor embodiment that matters, we found that observers react differently to visually similar drawings produced by agents with a different drawing style. Our findings show that sensitivity for differences in kinetic dynamics transfer from live movement to static traces of movement and enable an observer to discriminate between different motor origins of static traces. Observers are thus not only capable to discriminate between traces resulting from movement and lines not resulting from movement [29]–[30], but they are also sensitive to differences between motor origins. Even if kinematic differences in drawing style are very subtle, they must be responsible for the significant difference in percentage of correct detection of the motor origin. Possible explanations for the better recognition of unambiguous human drawings versus more ambiguous human and/or robot drawings point to the additional role of motor understanding over and above a purely visual understanding, whereas the less good scores for the ambiguous drawings could point to a possible confusion similar to the “uncanny valley” phenomenon.

The present study also shows a number of limitations that could be addressed in further studies. This study investigated the sensitivity to differences in the motor origin of drawings (robot or human) in the case where the human draughtsman is not free but constrained to the original drawing by the robot. This allowed us as much control over the independent variables as possible. We do not suspect that the kinematic qualities of the artists' drawings gestures were affected by the mechanic gestures of the robot, since the artists were not instructed to draw *like* robots or to reproduce the robot movements (which moreover they did not witness), but they were asked to replicate the elements of the drawing in there own drawing style. However, it would be interesting to see what happens if we change the direction of copying and have a set of human drawings redrawn by a robot, and have humans copy again such drawings. To what degree would this affect the observer's sensitivity to differences in motor origins of the drawings?
